# SuPepMem: A database of innate immune system peptides and their cell membrane interactions

**DOI:** 10.1016/j.csbj.2022.01.025

**Published:** 2022-01-31

**Authors:** Fabián Suarez-Leston, Martin Calvelo, Gideon F. Tolufashe, Alicia Muñoz, Uxía Veleiro, César Porto, Margarida Bastos, Ángel Piñeiro, Rebeca Garcia-Fandino

**Affiliations:** a Department of Organic Chemistry, Center for Research in Biological Chemistry and Molecular Materials, Santiago de Compostela University, CIQUS, Spain; b Departamento de Física Aplicada, Facultade de Física, Universidade de Santiago de Compostela, E-15782 Santiago de Compostela, Spain; c Departament de Química Inorgánica i Orgànica and Institut de Química Teòrica i Computacional (IQTCUB), Universitat de Barcelona, Barcelona 08028, Spain; d CIQUP, Centro de Investigação em Química, Departamento de Química e Bioquímica, Faculdade de Ciências, Universidade do Porto, Porto, Portugal

**Keywords:** Host defense peptides, Antimicrobial peptides, Membrane, Innate immune system, Molecular dynamics simulations, Database, Machine learning

## Abstract

Host defense peptides (HDPs) are short cationic peptides that play a key role in the innate immune response of all living organisms. Their action mechanism does not depend on the presence of protein receptors, but on their ability to target and disrupt the membranes of a wide range of pathogenic and pathologic cells which are recognized by their specific compositions, typically with a relatively high concentration of anionic lipids. Lipid profile singularities have been found in cancer, inflammation, bacteria, viral infections, and even in senescent cells, enabling the possibility to use them as therapeutic targets and/or diagnostic biomarkers. Molecular dynamics (MD) simulations are extraordinarily well suited to explore how HDPs interact with membrane models, providing a large amount of qualitative and quantitative information that, nowadays, cannot be assessed by wet-lab methods at the same level of temporal and spatial resolution. Here, we present SuPepMem, an open-access repository containing MD simulations of different natural and artificial peptides with potential membrane lysis activity, interacting with membrane models of healthy mammal, bacteria, viruses, cancer or senescent cells. In addition to a description of the HDPs and the target systems, SuPepMem provides both the input files necessary to run the simulations and also the results of some selected analyses, including structural and MD-based quantitative descriptors. These descriptors are expected to be useful to train machine learning algorithms that could contribute to design new therapeutic peptides. Tools for comparative analysis between different HDPs and model membranes, as well as to restrict the queries to structural and time-averaged properties are also available. SuPepMem is a living project, that will continuously grow with more simulations including peptides of different sequences, MD simulations with different number of peptide units, more membrane models and also several resolution levels. The database is open to MD simulations from other users (after quality check by the SuPepMem team). SuPepMem is freely available under https://supepmem.com/.

## Graphical abstract

**Figure:**
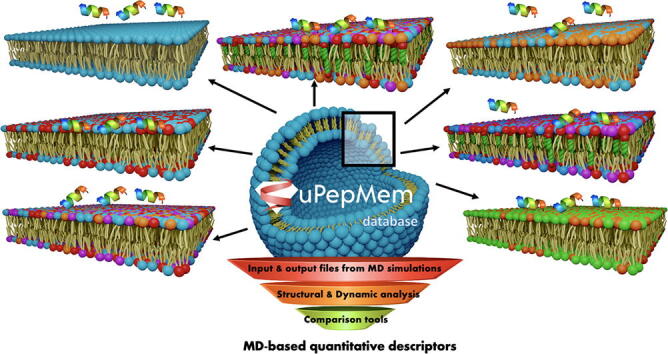

## 1 Introduction

Host defense peptides (HDPs), also called antimicrobial peptides (AMPs), are key components of what is called the innate immune system, present in all living organisms [1], [2]. They have been essentially associated to a defensive role against exogenous infections caused by bacteria, viruses and fungi. Thus, they are considered as powerful and versatile endogenous antibiotics, unsusceptible to bacterial resistance evolution for over millions of years [3]. Recent studies have pointed out the connection between HDPs and other pathologies, such as cancer and a range of inflammatory and autoimmune human diseases [4], [5], [6], linked by their action mechanism. Although HDPs lengths, sequences and 3D conformations differ significantly, most of these peptides share structural and physicochemical properties that allow them to target negatively charged membranes, such as those present in cancer cells and bacteria, but not in healthy mammalian cells, which are typically neutral. Thus, the purpose of these membrane-targeting HDPs is to recognize and disrupt strange lipid patterns likely caused by infections or by any other pathology. This action mechanism does not depend on the presence of protein receptors [7]. Lipid profile singularities have been found in cancer [8], [9], [10], [11], inflammation [12], bacterial and viral infections [13], and even in senescent cells [14], [15], [16]. This explains the presence of HDPs in all these pathologies, enabling the possibility to use them as therapeutic targets and/or diagnostic biomarkers. Furthermore, despite its protective skills, HDPs might also function as a ‘double-edged sword’ by triggering additional chronic inflammation, thus also damaging the host. Exacerbated autoimmune responses in infections, including COVID-19, have been attributed to the direct interaction of pathological membrane compositions with HDPs [13]. Overexpression of HDPs also contributes to aging through cytotoxic effects [17]. In fact, the dysregulation of their expression differentiates Alzheimer's disease from normal aging [18]. Intriguing yet contradictory findings have demonstrated that while some HDPs have anti-tumoral activity and are under-expressed in solid tumors, others are overexpressed and pro-tumorigenic [18]. This Dr. Jekyll/Mr. Hyde characteristic of HDPs paves the way to new treatment strategies in diseases affected by chronic inflammation based on the use of anti-HDPs to cancel out their undesired effects [19].

More than 3000 HDPs have been isolated from various cells and tissues of animals, insects, plants and bacteria [20], [21]. However, only a few of them have been transferred to the drug market so far [22]. Challenges toward clinical application of HDPs include cytotoxic effects, production costs, and problems related to sustained and targeted delivery or efficacy. Aiming to solve these problems, the synthesis of new artificial HDPs has been proposed [23]. The major barrier towards the design of these molecules is the poor understanding of their action mechanism as well as its connection with the peptide sequence and structure. The currently employed trial and error approach for the development of new active peptides [24] is highly inefficient, so better alternatives based on quantitative criteria are required. A comprehension of the action mechanisms of HDPs is essential to understand their role in a large range of pathologies where the lipid composition is significantly altered, such as cancer, immune disorders and even aging. Although descriptive models of the interaction between HDPs and cell membranes, such as pore-forming and carpet-like models, have already been published, atomic level resolution features have scarcely been reported [25], [26]. Molecular dynamics (MD) simulations are extraordinarily well suited to explore these events with high temporal and spatial resolution, providing a large number of quantitative descriptors [27], [28]. With this information, new strategies could be proposed to efficiently design new powerful therapeutic HDPs. Large sets of MD simulations including a variety of well characterized systems, together with an algorithm able to effectively analyze all this information, would contribute to solve this problem.

Here we present SuPepMem, a web-based database containing structural and dynamic information obtained from computational MD simulation trajectories of HDPs interacting with different membrane models. An effort to include representative quantitative descriptors has been done, to facilitate global analysis based on machine learning (ML) algorithms. The insights provided by this database are expected to make a significant contribution to ongoing efforts worldwide to design novel therapeutic and biomarkers HDPs derivatives as well as to understand the links between lipid-altered pathogenic states.

## 2 Materials and methods

### 2.1 Simulation systems and parameters

**Fig. 1:**
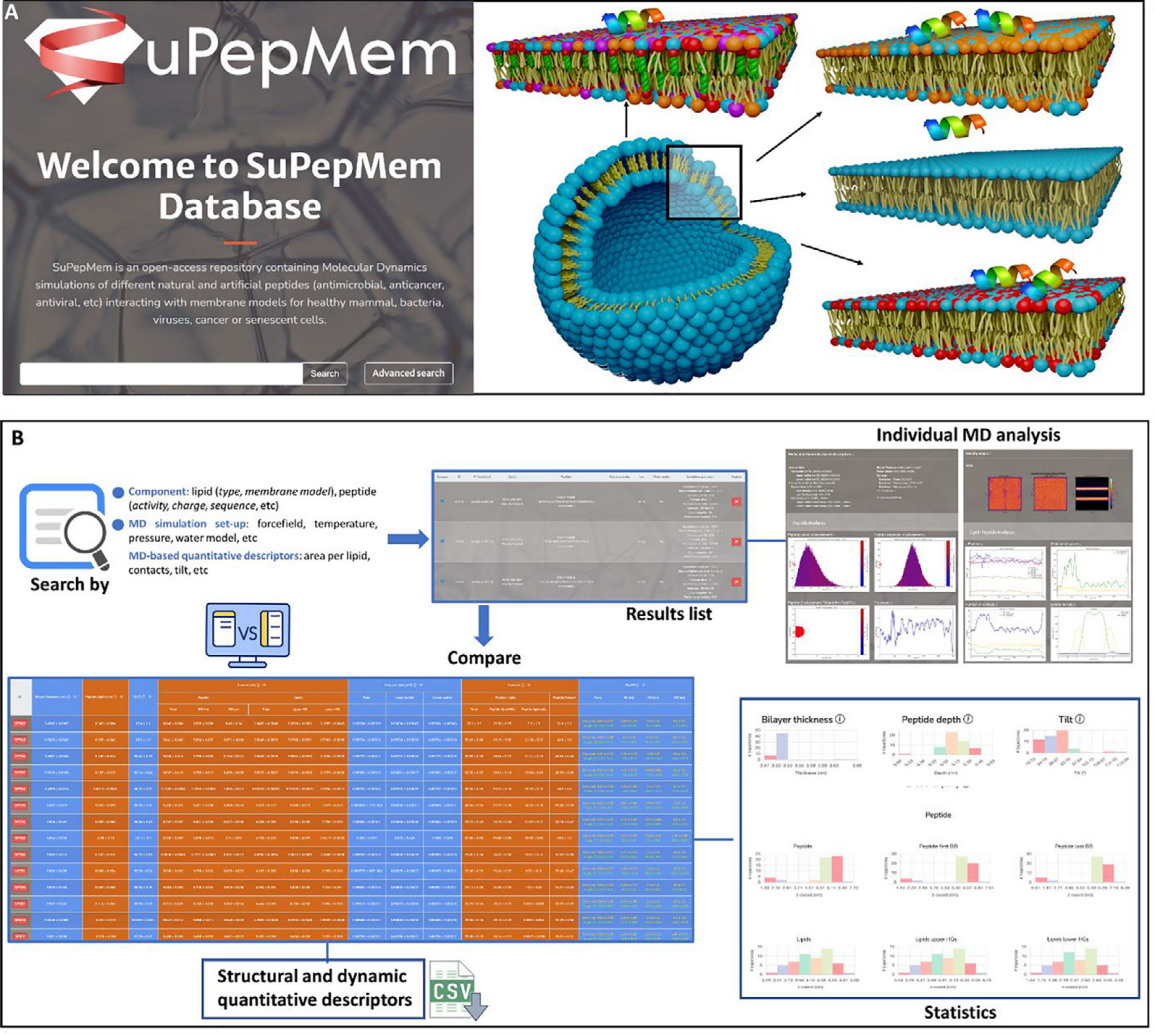
**A**. SuPepMem main page and schematic model of the interaction between HDPs with different membrane models. Each color represents a different lipid type. **B**. Schematic representation of the SuPepMem workflow.

SuPepMem has been designed to contain input files and analysis of MD simulation trajectories of peptides interacting with membrane models. Any force field, resolution, number of molecules, composition, geometry of the simulation box or of the membrane, and trajectory length can be considered. A set of more than 400 simulations have been incorporated in the current version of SuPepMem. They comprise 52 HDP sequences interacting with six different membrane models (Fig. 1A, Fig. S1 and Table S1): *i*) POPC (1-palmitoyl-2oleoyl-*sn*-glycero-3-phosphocholine), as a model of healthy mammal; *ii*) POPG (1-palmitoyl-2-oleoyl-*sn*-glycero-3-phosphoglycerol) and POPE (1-Palmitoyl-2-oleoyl-*sn*-glycero-3-phosphoethanolamine) in a relation POPG:POPE (3:1) as a model of Gram-positive bacteria; *iii*) POPG:POPE (1:3) as a model of Gram-negative bacteria; *iv*) POPG:POPE (1:9) as another model of bacteria; *v*) a model of realistic plasma membrane, where the outer monolayer is enriched in sphingomyelin (DPSM) and 1,2-dioleoyl-*sn*-glycero-3-phosphocholine (DOPC), while the inner monolayer is enriched in 1,2-dioleoyl-*sn*-glycero-3-phosphoethanolamine (DOPE) and 1,2-dioleoyl-*sn*-glycero-3-phospho-Lserine (DOPS) [27], *vi*) a model of a cancer membrane based on *v)* but with increased proportion of DOPS and DOPE in the extracellular leaflet [27].

The peptide sequences were selected from the DRAMP2.0 database [29], following a triple criteria: 1) significant membrane activity; 2) sequence length lower than 80 amino acids; and 3) likely ⍺-helix structure when the peptide is interacting with lipid membranes. The length, total charge, electrostatic and hydrophobic dipolar moment distributions of the HDPs currently available in SuPepMem can be visualized in Fig. S2. Three dimensional coordinates for all the peptide atoms, assuming ideal α-helices, were generated from the selected sequences using a homemade code based on the biopython and PeptideBuilder libraries [30], [31]. The resulting structures were then mapped into a MARTINI v2.2 coarse-grained (CG) model [32]. Although it is known that HDPs usually have random conformations in aqueous solution that fold into helix upon interacting with membranes, the initially formed ⍺-helical structures were preserved in our simulations by imposing artificial forces. This approach is not a requirement to include trajectories in SuPepMem but it was used for the simulations currently contained in the database in order to speed up the interaction between the peptides and the membranes. The introduction of atomistic resolution simulations without restrictions on the helical structures is planned for future stages of the development of the database. The CG structures for all the membrane models were generated using the MARTINI Builder module of the Charmm graphical-user interface [33]. Each membrane model, containing about 500 lipids distributed in 250 units per leaflet, was introduced in a 12.5x12.5x9.5 nm^3^ rectangular simulation box. The corresponding HDPs were placed with their center of mass at approximately 1 nm far from the membrane surface with the helical axis parallel to the membrane plane, randomly rotated around its own symmetry axis. The resulting boxes were solvated using standard or polarizable (depending on the system) MARTINI water molecules to simulate the aqueous environment. Afterwards, the counterions required to neutralize the electrostatic charge of the whole system were added. A steepest descent energy minimization was performed to remove the bad atomic coordinates overlaps and clashes, followed by 200, 500, and 2000 ps of stepwise constant pressure equilibrations using the Berendsen barostat [34]. The production trajectories were carried out at 1 bar and 300 K using the semiisotropic Parrinello–Rahman barostat [35], and a V-rescale thermostat [36]. The LINCS algorithm [37] was used to eliminate bond vibrations or its drifting in the system. The Particle Mesh Ewald [38] method with periodic boundary conditions was used to treat the long-range electrostatics with a 1.1 nm cut-off in direct-space. The dielectric constant was set to 2.5 when the polarizable force field was used and to 15 for the non-polarizable version of MARTINI [39]. Van der Waals interactions were computed using a spherical cut-off of 1.1 nm. Production trajectories of 5 μs with a time step of 25 fs for the integration of the motion equations were performed keeping only the constraints required to maintain the ⍺-helical structure of the peptides. Energies and positions of atoms were stored every 1000 steps (25 ps). All MD simulations were performed with the GROMACS 2018.3 software package [40]. The topologies, coordinates and input software files (with top, itp, gro and mdp extensions for GROMACS) required to perform the simulations are directly available from the database.

### 2.2 Analysis of the initial structures

**Fig. 2:**
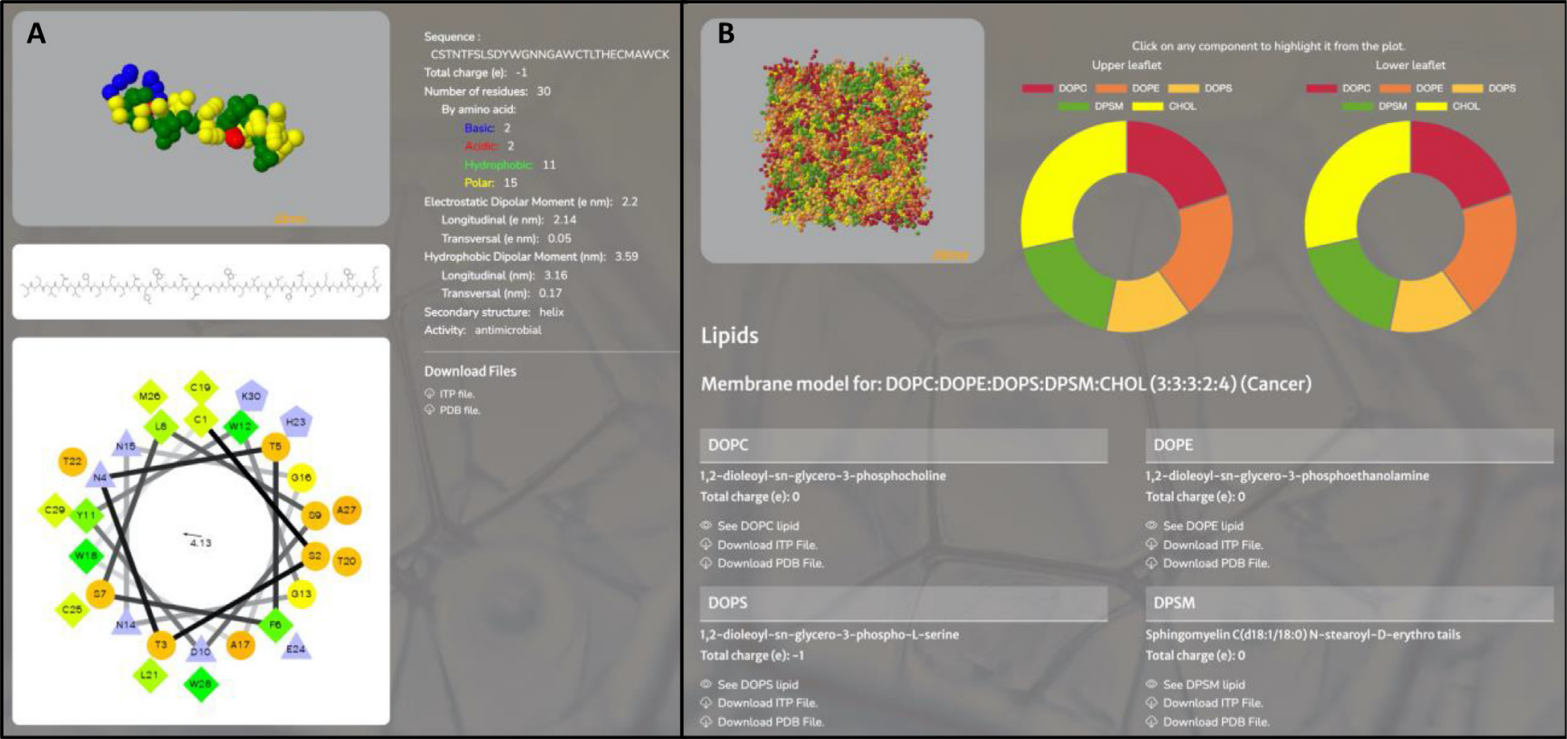
**A**. Example of an analysis of the initial structure of one of the peptides included in SuPepMem database. Helical wheel representation [41], 2D, and 3D representations of each peptide are shown, together with the sequence, number and type of residues, total charge and electrostatic and hydrophobic dipolar moments with their longitudinal and transversal components. The topology and coordinate files of the peptides in GROMACS format can be directly downloaded. **B**. Analysis of the initial structure of one of the membrane models included in SuPepMem database, corresponding to a cancer model. The composition of the membrane is represented indicating the name, structure and charge for each type of lipid per leaflet. The topology and coordinate files of the corresponding lipids in GROMACS format can be directly downloaded.

Different analyses on the initial structures of the HDPs and the corresponding membranes for each simulation were performed (Fig. 2). Specifically, helical wheel, 2D, and 3D representations of each peptide are shown, together with the sequence, number and type of residues, total charge and electrostatic and hydrophobic dipolar moments with their longitudinal and transversal components (Fig. 2A). The composition of the membrane is also represented indicating the name, structure and charge for each type of lipid per leaflet (Fig. 2B).

### 2.3 Analysis of the MD trajectories

The analysis of the simulations was carried out using GROMACS tools and specific code written by us in Python mainly based on the MDAnalysis [42], NumPy [43] and Matplotlib libraries [44] . In brief, the average values of several properties along the last microsecond of the simulations were determined including: the area per lipid and per leaflet; the Z coordinate of the peptide center of mass as well as those of the first and last residue; the Z coordinate of the lipid head groups center of mass; the bilayer thickness; the peptide insertion into the bilayer (penetration length of the center of mass of the peptide in the membrane); the number of contacts (using a cut-off of 0.6 nm) between the peptide backbone and the water molecules, lipid head and lipid tail groups; the angle between the helical axis of the peptide and the normal to the membrane (tilt); and the peptide lateral and rotational displacements using time windows of 5, 50 and 200 ns. These average values are extremely important since they are MD based quantitative descriptors that could be used to assess the sensitivity of the peptides to different membrane compositions. Note that the Z coordinate is also key since the membrane surface is contained in the XY plane and the Z coordinate determines the relative location of the peptide with respect to the lipid bilayer. The peptide insertion is the closest distance between the center of mass of the peptide and the membrane surface. Negative values of this property are obtained when the peptide is between both leaflets. All these properties with their standard deviation can be downloaded from SuPepMem in JSON format for further external analysis. The plots of all these properties as a function of time are also provided in the database in order to visually observe how they evolve along the trajectories as well as the strength of the fluctuations. For the lateral and rotational displacements, distribution plots with a color gradient for the time are employed, and a map in the lateral vs rotational displacement 2D space (the peptide displacement fingerprint - PepDF) is also generated. Density maps per leaflet are also included for each lipid consisting the membrane models. Finally, the number of contacts per residue (also considering the backbone and a cut-off of 0.6 nm) between each type of amino acid of the peptide and each type of lipid in the bilayer is determined and plotted as a stacked histogram. These results are accompanied by an interactive 3D visualization of the final frame extracted from the trajectory.

### 2.4 Database technical details

The structure of SuPepMem is prepared to store systems of any number of molecules and composition, different force fields and resolution levels, and any geometry of the simulation box or of the membrane model (flat membranes or vesicles). The transfer of all the information from the input and trajectory files to the database is fully automated, including the determination of quantitative descriptors and the generation of the plots with dynamic information. These scripts are currently compatible only with GROMACS files, but they are easy to adapt for other popular MD packages. A first set of 403 systems resulting from the combination of 52 HDPs and 6 different membranes models, simulated using the MARTINI V2.2 force field for 5 μs each has already been transferred to SuPepMem.

All data are stored in a database, using the structured query language (SQL) to ensure portability. The website is created with HTML5 and PHP for the server side operations and Javascript for the client side to represent the chemical structures and graphics. SuPepMem uses JSmol (an open-source Java viewer for chemical structures in 3D, http://www.jmol.org) to show the molecular structures of the peptides and the membranes.

## 3 Results

SuPepMem provides a rapid and user-friendly online database access with multiple functionalities:

### 3.1 Simple and Advanced search

**Fig. 3:**
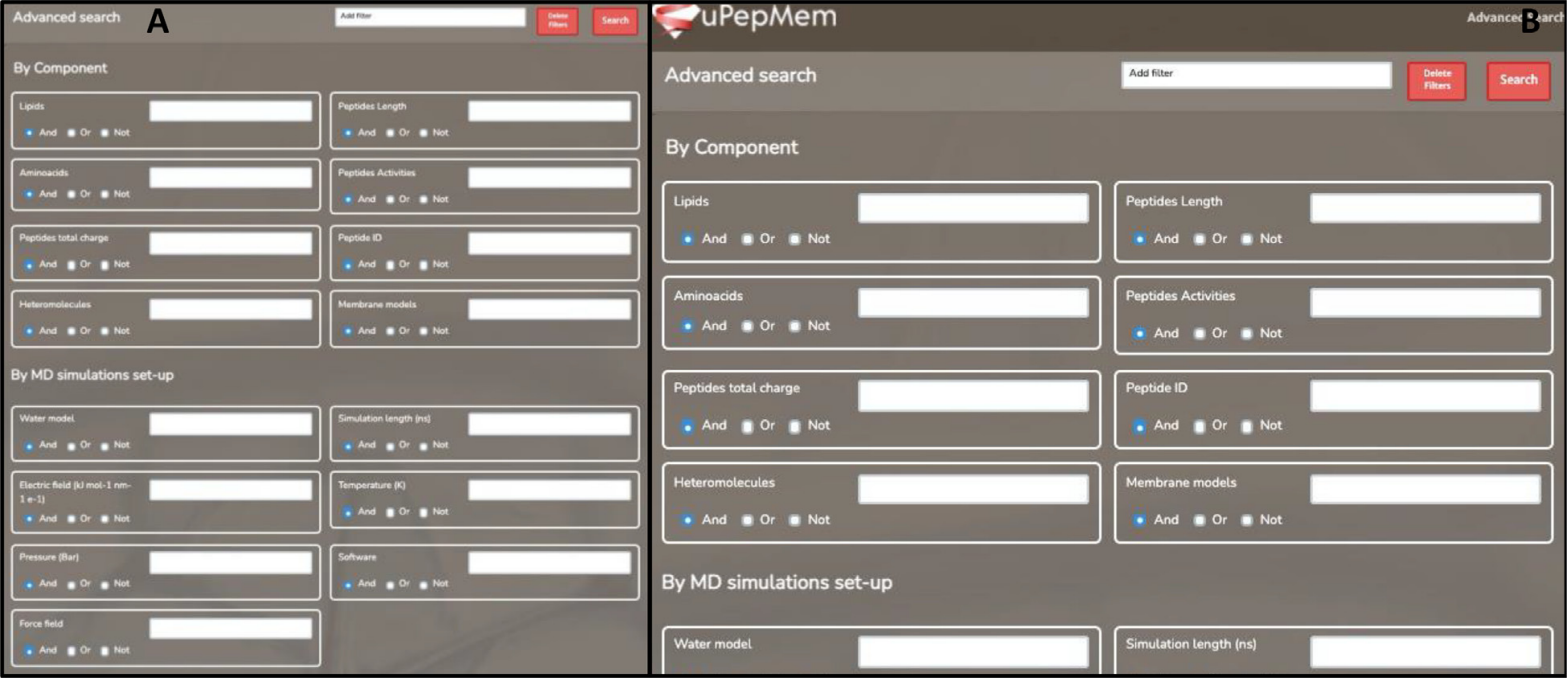
Screenshot of the Advanced Search functionality. It is possible to filter trajectories as a function of many parameters including (**A**) components of the systems (name, sequence or structural properties of the peptides or lipids) or parameters of the MD simulation (water model, electric field, pressure, temperature, force field, simulation length and MD software used). **B**. It is also possible to search by property obtained from the analysis of the interaction between the peptides and the membrane models (MD-based quantitative descriptors). More filters can be added (or deleted).

Simple Search allows to find MD trajectories containing a specific segment of a peptide sequence (e.g. GWLIR), a peptide ID (e.g. DRAMP00008) or a lipid type (e.g. POPC). For more elaborated selections, the user is invited to make use of the Advanced Search (Fig. 3). This functionality opens up a much wider range of filtering possibilities, which can be combined using the AND/OR logical operators to easily find a particular case of interest: *i*) by component (aminoacids, peptide ID, peptide activity, peptide length, peptide total charge, lipid name, membrane model and the presence or not of heteromolecules in the membrane) (Fig. 3A); *ii*) by MD simulations set-up (water model, electric field, pressure, temperature, force field, simulation length and MD software used) (Fig. 3A); *iii*) by MD-based quantitative descriptors (area per lipid, bilayer thickness, contacts between different parts of the peptide and the lipids, Z coordinate of different regions of the peptide and the membrane, peptide insertion and peptide tilt) (Fig. 3B). The user can also incorporate more filters (i. e. ions, temperature or pressure coupling, number of particles, supercomputer, etc) through the “Add filter” option. The results of the specific query are visualized online and can also be downloaded in CSV format (Fig. S3).

### 3.2 MD simulations input/output files for download

All topologies, coordinates and input files (with top, itp, gro and mdp extensions for GROMACS) required to set-up and perform the MD simulations are directly available from the database. In that way, the user has access to all the technical options that have been used for the MD simulations, and can reproduce or personalize them in local computers. Thus, SuPepMem serves also as a public repository for structural and force field parameters of an increasing number of peptides and membrane models of different pathologic states (mammalian, cancer, bacterial, senescent cells, etc).

### 3.3 Analysis of individual MD trajectories

**Fig. 4:**
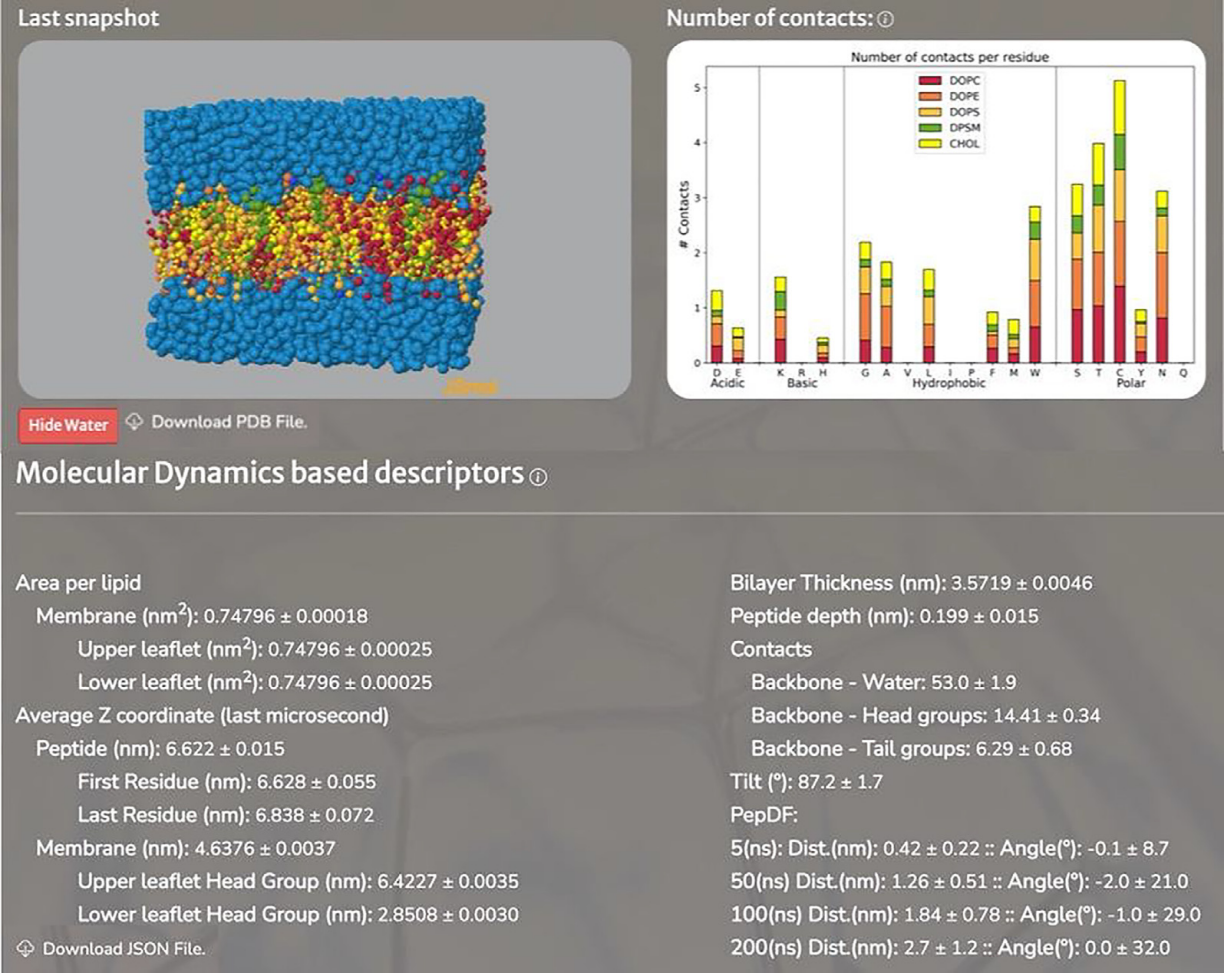
Example of the analysis of an individual trajectory providing MD-based quantitative descriptors for different properties: area per lipid, bilayer thickness, the Z coordinates of the different component of the system, the number of contacts between the different type of residues and lipids, the angle between the helical axis and the normal to the membrane plane (tilt) and peptide lateral and rotational displacement distributions at different time windows (PepDF). All these descriptors can be downloaded into a JSON file. A graphic representation of different analyses extracted from the trajectory can be also visualized, organized in different subsections: Peptide analyses, lipid analyses and lipid-peptide analyses (Fig. S4-S6).

The results of the Simple or Advanced Search are displayed on the screen, headed by a summary of the main characteristics of the simulated system, and divided in three different sections: *i*) *Peptide* (Fig. 2A), containing its total charge, number and type of residues, electrostatic and hydrophobic dipolar moments (longitudinal and transversal components in each case), secondary structure, 3D structure (interactive by JSmol), 2D-chemical structure, helical wheel representation, and topology/coordinates downloadable files; *ii*) *Membrane* (Fig. 2B), where the composition of each monolayer is represented in an interactive circular plot, together with the specific details for each lipid type and the pdb and itp downloadable files for the model membrane used; and *iii*) *Analysis* (Fig. 4), enclosing the last snapshot of the trajectory (interactive by JSmol), together with a graphic representation of different analyses extracted from the whole trajectory or from the last microsecond, depending on the analysis type, organized in different subsections (Fig. S4–S6):

• **Peptide Analyses**: tilt, lateral and rotational displacement, and their correlation into what we have called Peptide Displacement Fingerprint – PepDF.
• **Lipid Analyses**: area per lipid and also different density maps
• **Lipid-Peptide Analyses**: average z-position for the peptide, membrane, first and last backbone residue and upper or lower lipid headgroups; minimum distance between the peptide backbone and the water or the lipid headgroups and tailgroups; number of contacts between the peptide and the water or the lipids separated by lipid headgroups or lipid tails; and lateral density for the lipid headgroups, tailgroups and the peptide. A plot with the number of contacts between the different types of peptide residue and each lipid type in the membrane is also represented. Besides the graphical analysis, one the most valuable features of SuPepMem is that it also provides a list of MD-based quantitative descriptors for all these different properties, which can be downloaded into a JSON file, for further analysis outside of the database (Fig. 4).

### 3.4 Tools for comparative analysis between different HDPs and model membranes

SuPepMem includes tools to easily compare the results of a particular query, or just for the particular selections made by the user. The results of the comparative analysis consist of a tabulated list of quantitative descriptors from MD simulations that can be downloaded in CSV format (Fig. S7): bilayer thickness, peptide insertion, peptide tilt, Z coordinate for different parts of the peptide and the lipids, area per lipid (total and for each leaflet), contacts between different parts of the peptides, the lipids and the solvent and the peptide fingerprint defined by us as PepDF). These MD-based quantitative descriptors can be directly employed to train ML algorithms and/or directly analyzed through statistical tools incorporated in SuPepMem (see below).

### 3.5 Statistical data analysis

All the data contained in SuPepmem can be visualized through different statistic representations, focused on the membrane model, the peptide activity, the peptide length and the peptide electrostatic dipolar and hydrophobic moment. By clicking on the pie charts and on the bar graphs it is possible to directly obtain both the peptides/membranes that are included in the statistic plots an also the results from the database involving those systems. Furthermore, as it was commented before, a statistical analysis is also offered for the comparison of the results of a particular query made by the user (Fig. S8).

## 4 Discussion

SuPepMem is a public database designed to provide both the input files required to execute MD simulations of peptides interacting with membrane models and the analysis of the corresponding trajectories, with special attention to quantitative descriptors (Fig. 1B). The aim is to make public a large set of data that can be used to understand the action mechanism of HDPs as well as to design new therapeutic membrane-targeting peptides. The information contained in the database has been distributed in such a way that it is possible to filter trajectories as a function of many parameters including the name, sequence or structural properties of the compounds (peptides or lipids), the composition of the system and even some of the properties obtained from the analysis of the interaction between the peptides and the membrane models (Fig. 3). The web application allows filtering the available simulations by combining restraints in any of these parameters as well as to download the structural and technical descriptors for the resulting query in CSV format (Fig. S3). The main properties available for the user are the sequence, total charge, electrostatic and hydrophobic dipolar moments of the peptide, the lipid composition of each leaflet in the membrane model (thus explicitly considering the eventual presence of asymmetric membranes).

The analysis of each individual trajectory provides the last snapshot of the trajectory, interactive by JSmol, together with a list of dynamic quantitative descriptors for different properties, such as area per lipid, bilayer thickness, the Z coordinates of the different component of the system, the number of contacts between the different type of residues and lipids, the angle between the helical axis and the normal to the membrane plane (tilt) and peptide lateral and rotational displacement distributions at different time windows (PepDF) (Fig. 4). All these descriptors can be downloaded into a JSON file. The graphical representation of these parameters is also presented in different sections: Peptide Analyses, Lipid Analyses (including also density maps) and Lipid-Peptide Analyses (Fig. S4-S6).

Tools for comparative analysis between different HDPs and model membranes are also available. This allows, for instance, to compare the results of peptides of a certain length for different membrane compositions, or to assess how certain property depends on the transversal component of the hydrophobic moment of the peptide. This tool is expected to be increasingly useful as more data are added to the repository. The result of the comparison consists of a table containing all the dynamic quantitative descriptors for different properties of all the selected trajectories in the previous window, which can also be downloaded in CSV format for further analysis (Fig. S3). Furthermore, a statistical analysis from this table is offered to the user, to facilitate a visual comparison of the selection for the resulting query (see example in Fig. S8).

SuPepMem is open to accept simulations from any user, upon previous validation by our team members. This aims to facilitate the growth of the project. Simultaneously, we will also contribute with new trajectories. Currently, a set of membrane-inactive peptides are being simulated in order to have negative controls from which it is possible to learn. Additionally, CG simulations with several peptide units and atomic resolution simulations are being executed.

Thus, systematic information on how HDPs from the innate immune defense are able to distinguish lipid bilayers of different compositions, mimicking both pathologic and healthy cell membranes as a function of the structure of the HDPs and the membrane, will be key to understand how they work, as well as to train ML algorithms, aimed to design new therapeutic structures.

## CRediT authorship contribution statement

**Fabián Suárez:** Code development for analysis of the MD simulations and database backend. **Martin Calvelo:** MD simulations setting-up, execution and analysis. **Gideon F. Tolufashe:** MD simulations setting-up and execution. **Alicia Muñoz:** Code development for analysis of the MD simulations. **Uxía Veleiro:** Code development for analysis of the MD simulations. **César Porto:** Front-end development. **Margarida Bastos:** Writing – review & editing. **Ángel Piñeiro:** Conceptualization, Writing – review & editing, Supervision. **Rebeca Garcia-Fandino:** Conceptualization, Writing – review & editing, Supervision.

## Declaration of Competing Interest

The authors declare that they have no known competing financial interests or personal relationships that could have appeared to influence the work reported in this paper.
