# Supplementary material for: SuPepMem: A database of innate immune system peptides and their cell membrane interactions
Source: Comput Struct Biotechnol J. 2022 Jan 31;20:874–81. doi: 10.1016/j.csbj.2022.01.025 (PMC8844400; doi:10.1016/j.csbj.2022.01.025)
Supplement: Supplementary data 1 [file mmc1.docx]

SuPepMem: a database of innate immune system peptides and their cell membrane interactions

*Fabián Suárez,^[[1]](#footnote-1),2‡^ Martin Calvelo,^1,3‡^ Gideon F. Tolufashe,^4‡^ Alicia Muñoz,^2^ Uxía Veleiro,^2^ César Porto,^1^ Margarida Bastos,^4^ Ángel Piñeiro,^2^* Rebeca Garcia-Fandino^1,4^**

^1^Department of Organic Chemistry, Center for Research in Biological Chemistry and Molecular Materials, Santiago de Compostela University, CIQUS, Spain.

^2^Soft Matter & Molecular Biophysics Group, Department of Applied Physics, Faculty of Physics, University of Santiago de Compostela, Spain.

^3^Departament de Química Inorgánica i Orgànica and Institut de Química Teòrica i Computacional (IQTCUB), Universitat de Barcelona, Barcelona 08028, Spain.

^4^CIQUP, Centro de Investigação em Química, Departamento de Química e Bioquímica, Faculdade de Ciências, Universidade do Porto, Porto, Portugal.

*Corresponding author. E-mail addresses: [Angel.Pineiro@usc.es](mailto:Angel.Pineiro@usc.es) and [rebeca.garcia.fandino@usc.es](mailto:rebeca.garcia.fandino@usc.es)

**Supporting Information**

**Table S1**. Lipid composition of the monolayers of simulated membranes, models vi) and vii).

| **Component** | **Normal** | **Cancer** |
| --- | --- | --- |
|  | **Upper Lower** | **Upper Lower** |
| DPSM | 66 16 | 46 46 |
| DOPC | 70 37 | 50 50 |
| DOPE | 37 70 | 50 50 |
| DOPS | 0 50 | 33 33 |
| CHOL | 77 77 | 71 71 |

**Figure S1**. Membrane models currently used in SuPepMem database. From <https://supepmem.com/statistics>.


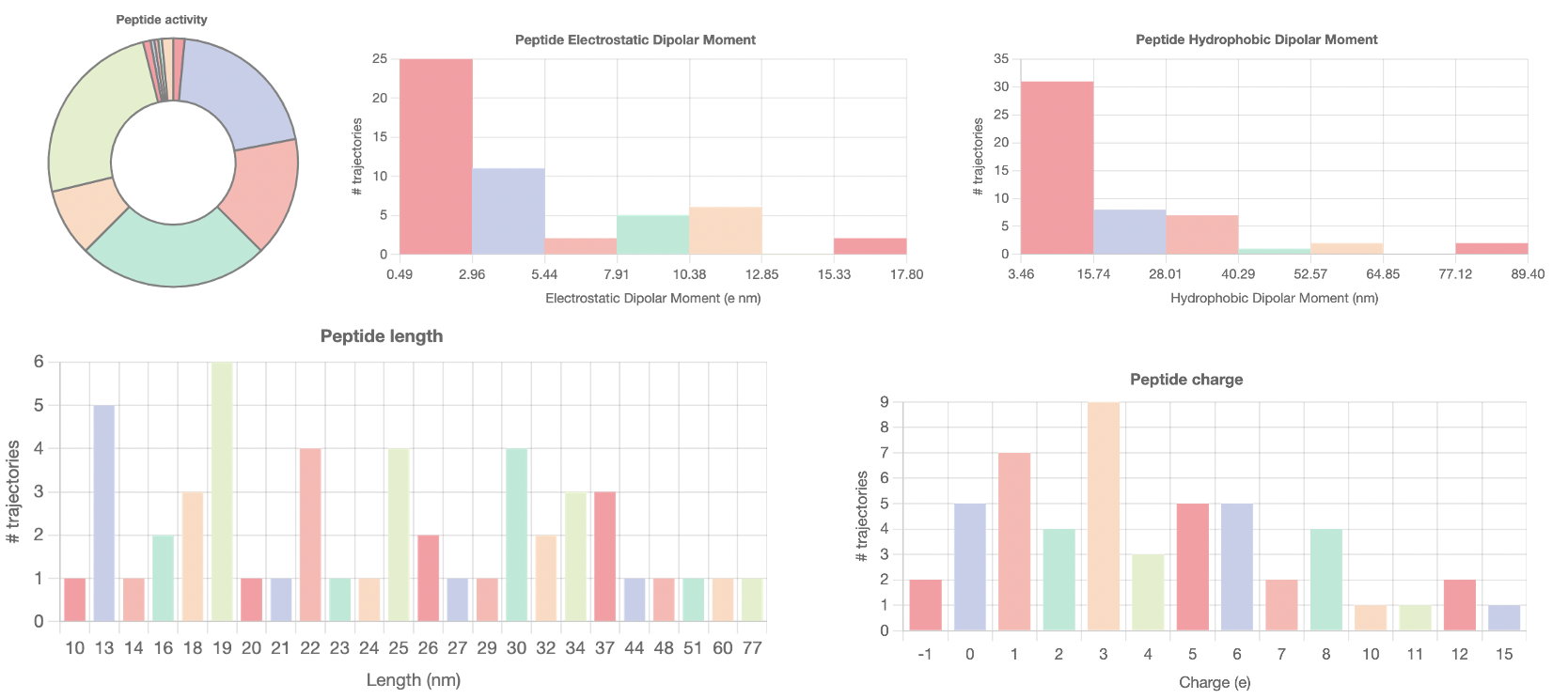


**Figure S2**. Characteristics of the HDPs included in SuPepMem database. Peptide activity, Peptide electrostatic dipolar moment, Peptide hydrophobic moment, peptide length, peptide charge. From: <https://supepmem.com/statistics>


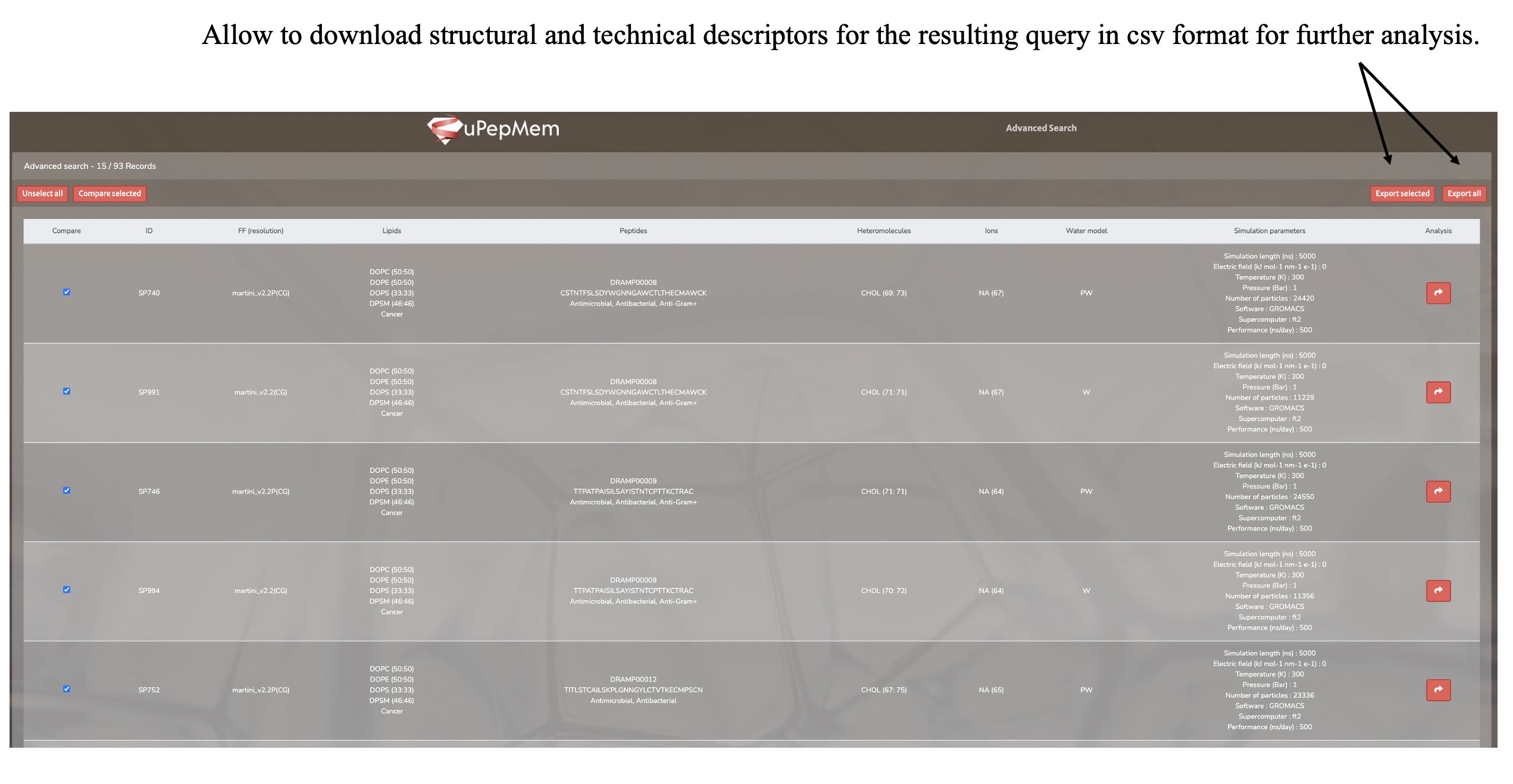


**Figure S3**. Detail of the buttons to download structural and technical descriptors for the resulting query in CSV format for further analysis.


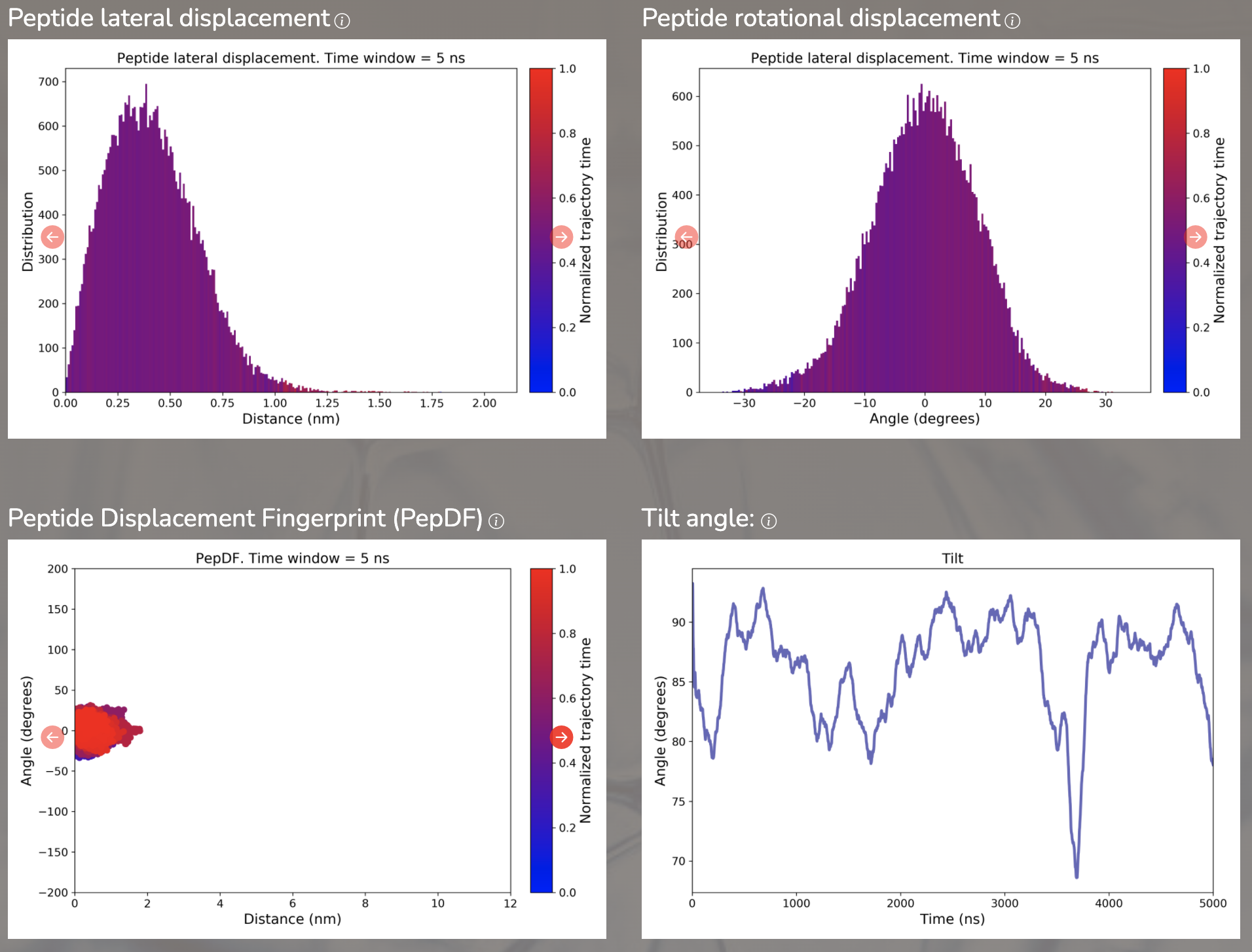


**Figure S4**. Example of the graphical representation of the analysis of a trajectory presented in the section “*Peptide Analyses*”. For more details, see: <https://supepmem.com/trayectorias/740>


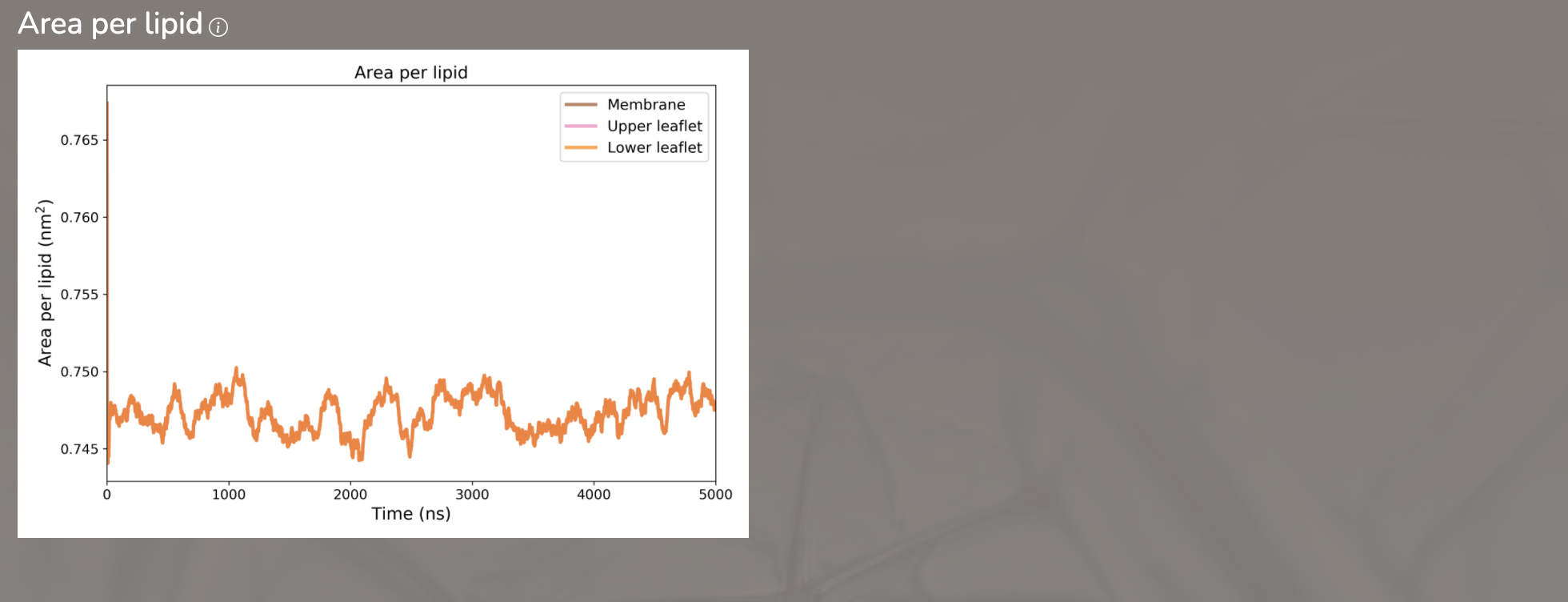


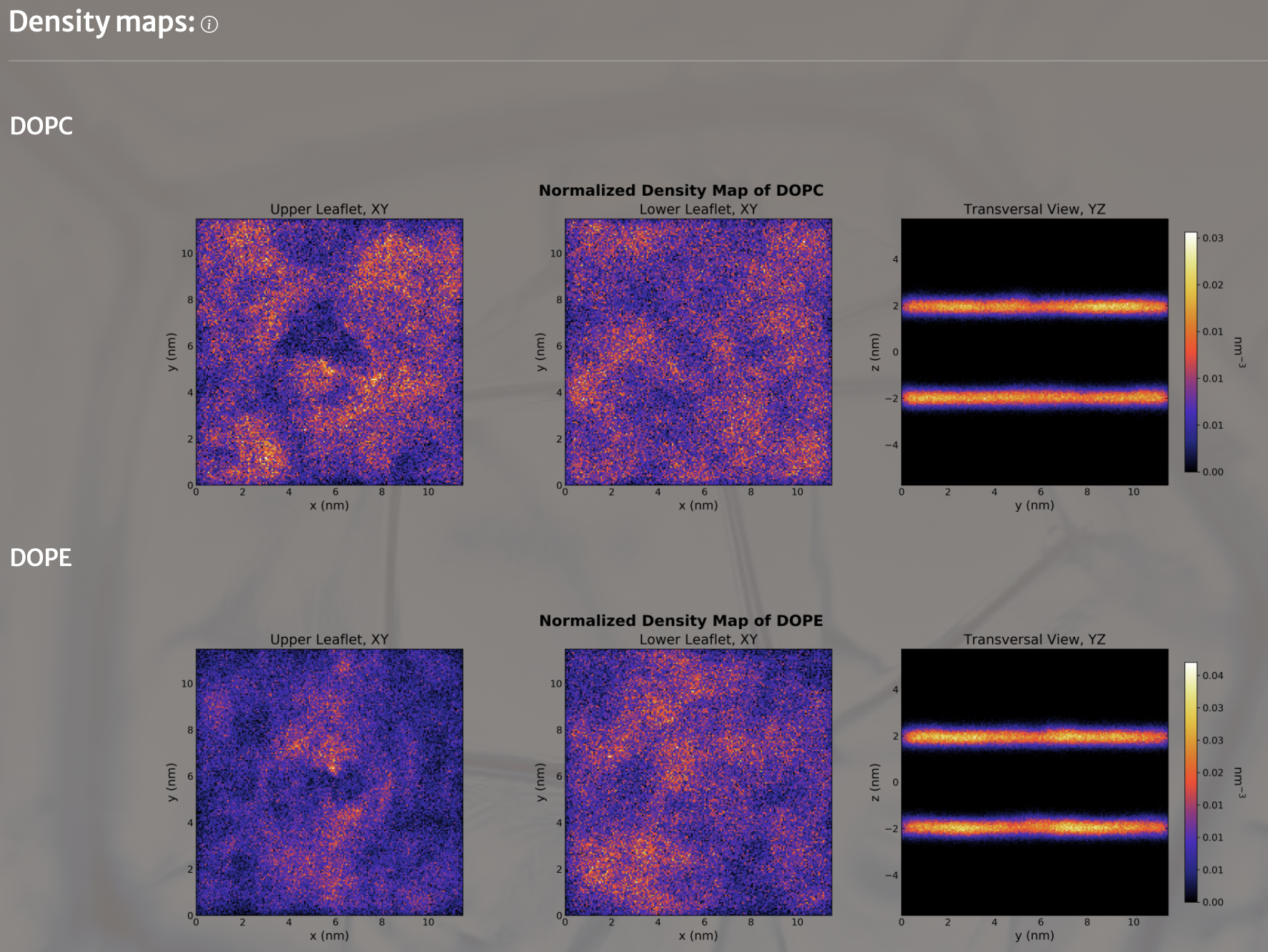


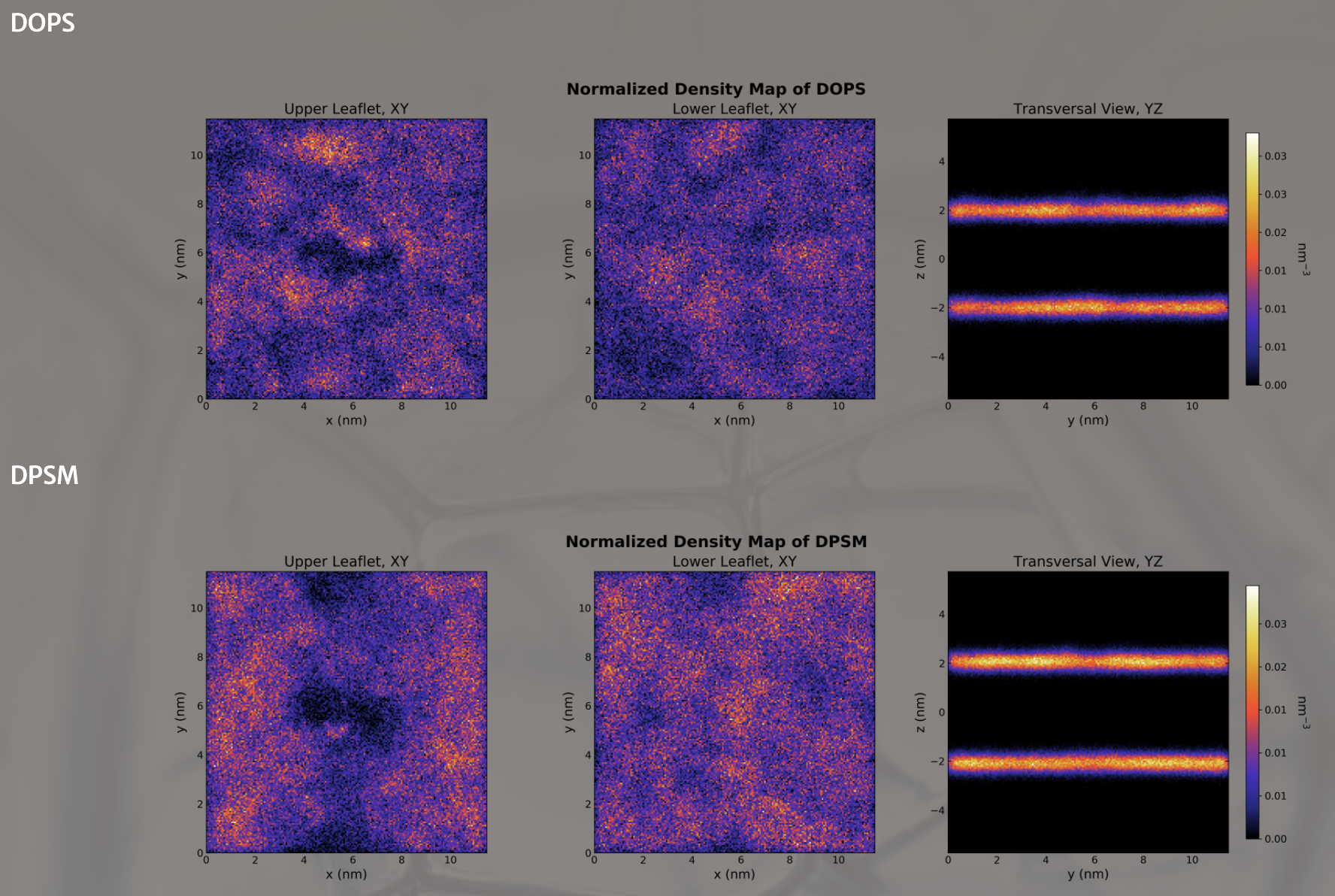


**Figure S5**. Example of the graphical representation of the analysis of a trajectory presented in the section “*Lipid Analyses*”. For more details, see: <https://supepmem.com/trayectorias/740>


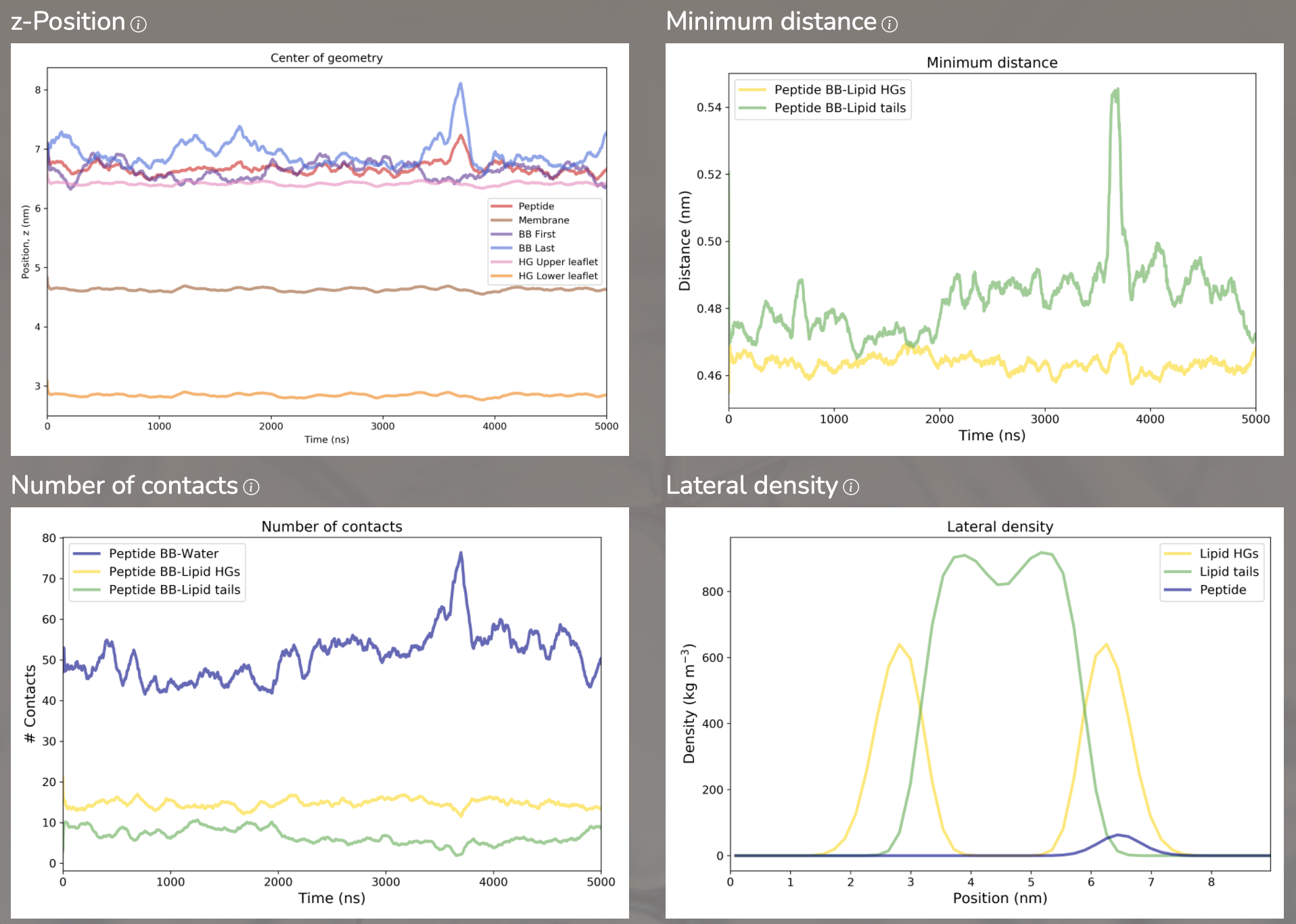


**Figure S6**. Example of the graphical representation of the analysis of a trajectory presented in the section “*Lipid-peptide Analyses*”. For more details, see: <https://supepmem.com/trayectorias/740>


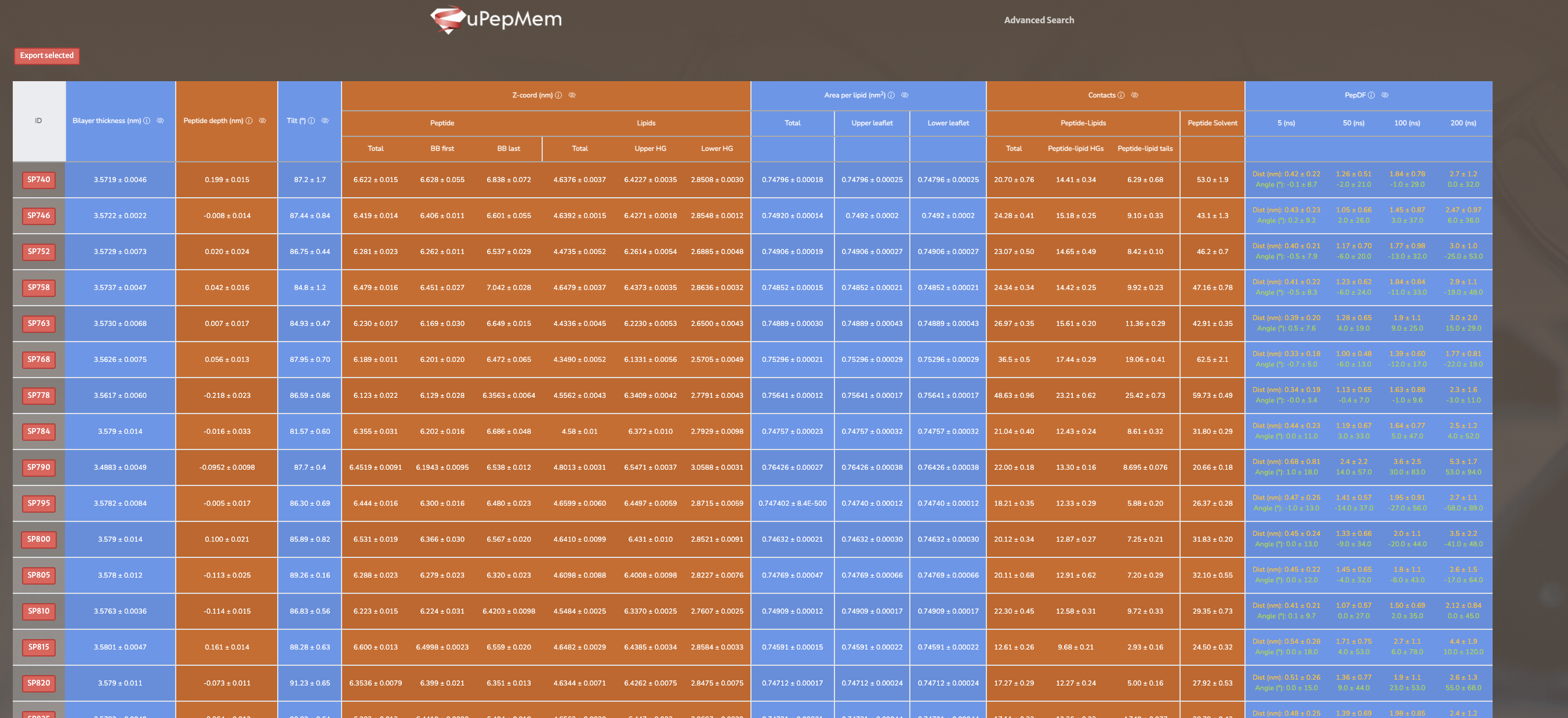


**Figure S7**: Example of the tabulated list of quantitative descriptors from MD simulations that can be downloaded in CSV format.

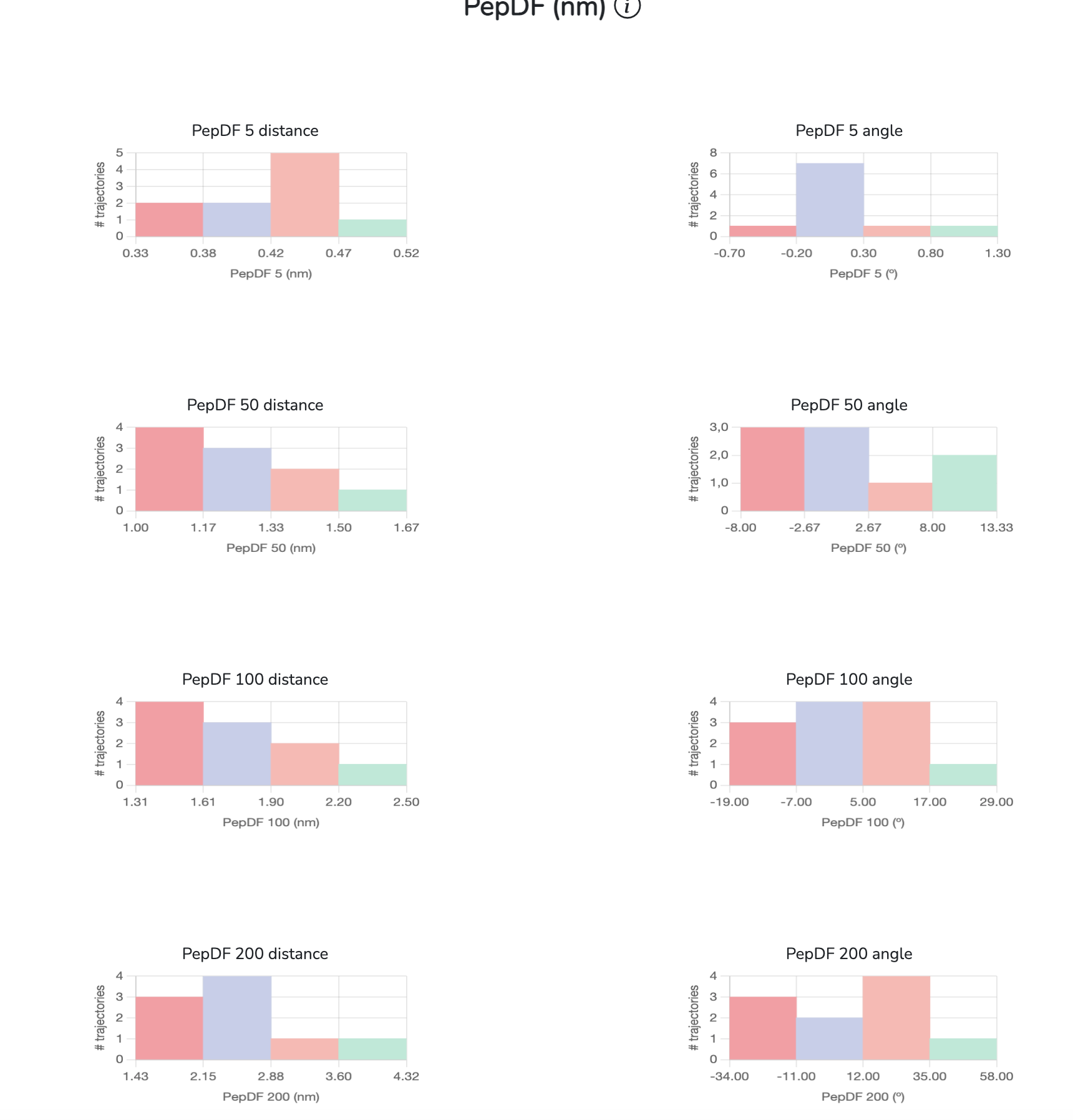


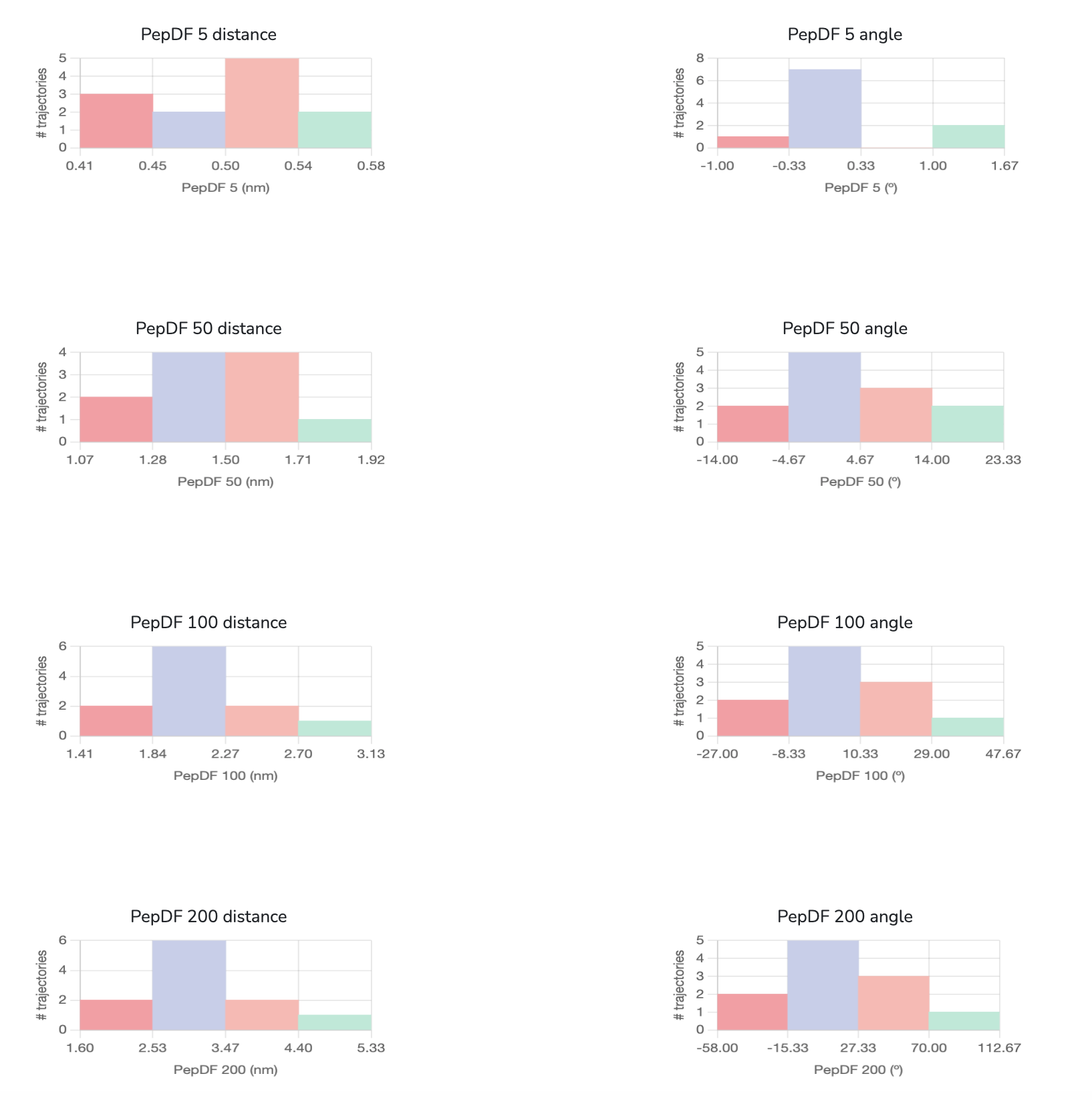


**Figure S8**: Example of statistical analysis from the data selected by the user using the comparative tool, to facilitate a visual comparison of the selection for the resulting query. An example is presented comparing the results obtained for peptides with charge +8 with neutral peptides (in dashed square) in a cancer membrane. It can be noticed, for instance, that the peptide insertion is larger in the case of the charged peptides, also correlated with a larger number of contacts between the charged peptides and the different parts of the membrane.

1. [↑](#footnote-ref-1)
